# Efficacy of esketamine for the treatment of postpartum depression and pain control following cesarean section: a randomized, double-blind, controlled clinical trial

**DOI:** 10.1186/s12871-024-02436-6

**Published:** 2024-02-06

**Authors:** Shurong Li, Zhifang Zhuo, Renwei Li, Kaikai Guo

**Affiliations:** 1 Department of anesthesiology, The First Hospital of Putian City, Putian, Fujian China; 2 Department of pain medicine, The First Medical Center, Chinese PLA General Hospital, Beijing, China

**Keywords:** Esketamine, Cesarean section, Patient-controlled intravenous analgesia, Postpartum depression, Postpartum women

## Abstract

**Background:**

Postpartum depression (PPD) following a cesarean delivery is a frequently seen complication. Despite the prophylactic effects of ketamine, the impact of esketamine on PPD in women undergoing cesarean section remains uncertain. This study aimed to assess the effectiveness of esketamine as an adjunct to patient-controlled intravenous analgesia (PCIA) in preventing PPD in women undergoing caesarean section.

**Methods:**

A total of 275 parturients undergoing caesarean section and subsequent patient-controlled intravenous analgesia (PCIA) were randomly assigned to receive either the control treatment (sufentanil 2 µg/kg + tropisetron 10 mg) or the experimental treatment with additional esketamine (1.5 mg/kg). The primary outcome measured was the incidence of postpartum depression (PPD), classified by Edinburgh Postnatal Depression Scale (EPDS) scores equal to or greater than 13 indicating PPD. Secondary outcomes included cumulative sufentanil consumption during specific time periods (0–24 h, 24–48 h, and 0–48 h) after the surgical procedure and numerical rating scale (NRS) scores at rest and during movements.

**Results:**

The final analysis included a total of 246 postpartum women who had undergone caesarean delivery. On postoperative day 42, the incidence of depression among the control group was 17.6%, which was significantly higher compared to the esketamine group with a rate of 8.2% (*P* = 0.02). The EPDS scores also showed a significant difference between the two groups, with a mean score of 9.02 ± 2.21 in the control group and 6.87 ± 2.14 in the esketamine group (*p* < 0.0001). In terms of pain management, the esketamine group showed lower sufentanil consumption in the 0–24 h (42.5 ± 4.58 µg vs. 50.15 ± 5.47 µg, *P* = 0.04) and 0–48 h (87.40 ± 9.51 µg vs. 95.10 ± 9.36 µg, *P* = 0.04) postoperative periods compared to the control group. Differences in movement were also observed between the two groups at 24 and 48 h after the cesarean Sect. (3.39 ± 1.57 vs. 4.50 ± 0.80, *P* = 0.02; 2.43 ± 0.87 vs. 3.56 ± 0.76, *P* = 0.02). It is worth noting that the frequency of side effects observed in both groups was comparable.

**Conclusions:**

Esketamine at a dose of 1.5 mg/kg, when used as a supplement in PCIA, has been shown to significantly reduce the occurrence of PPD within 42 days. Additionally, it has been found to decrease cumulative consumption of sufentanil over a 48-hour period following cesarean operation, all without increasing the rate of adverse effects.

**Trial registration:**

Registered in the Chinese Clinical Trial Registry (ChiCTR2200067054) on December 26, 2022.

## Introduction

Postpartum depression (PPD) is a prevalent psychological condition seen in females during the perinatal phase, characterized by a frequency ranging from 10 to 20%. Notably, the prevalence of PPD tends to be greater in middle and low-income nations [1]. PPD has been seen appear within a range of timeframes after childbirth, including as early as 6 weeks postpartum or later at 3, 6, 12, and even up to 36 months after delivery [2, 3]. Symptoms associated with PPD include feelings of sadness, a persistent state of low mood, diminished appetite, decreased interest in previously enjoyed activities, psychomotor retardation, and disturbances in sleep patterns. Severe PPD has been shown to be associated with an increased risk of suicidal thoughts [4]. PPD has negative effects on both newborns and family connections. Additionally, it places a substantial demand on medical services and resources [5, 6]. The effectiveness of pharmaceutical therapies for PPD is restricted owing to their delayed start of action and poor treatment effectiveness, despite their frequent use [7, 8]. Furthermore, it is worth noting that there are now no pharmacological interventions available for PPD.

Ketamine, a dissociative anesthetic, functions as an antagonist of the N-methyl-D-aspartate receptor (NMDAR), which can elicit prompt and enduring antidepressant properties in individuals with depression who have not responded to conventional treatments [9, 10]. Nevertheless, the utilization of ketamine in a therapeutic setting as an antidepressant is constrained by its adverse effects, which include dissociative and psychotomimetic manifestations, as well as the potential for addiction [11–13]. The anesthetic impact of esketamine, a ketamine dextral resolution, has been shown to be twofold more than that of the (R, S)-ketamine mixture and nearly thrice greater than that of (R)-ketamine [14]. Additionally, the incidence of adverse effects associated with esketamine was found to be decreased compared to subanesthetic dosages of ketamine [15].

Multiple clinical investigations have provided evidence supporting the notion that the perioperative intravenous (IV) injection of ketamine has the potential to decrease the occurrence of PPD [16, 17]. The administration of esketamine by IV means resulted in prompt and persistent antidepressant impacts in individuals with depression [17, 18]. Wang and colleagues (2022a) demonstrated that when esketamine was used in combination with sufentanil for patient-controlled intravenous analgesia (PCIA), the overall usage of sufentanil decreased [19]. However, in a randomized controlled trial (RCT), researchers discovered that the use of esketamine for intraoperative pain management and as an adjunct in PCIA did not lead to a decrease in the incidence of PPD risk among women who underwent elective cesarean Sect. [16]. Therefore, the current pool of data is considered insufficient due to significant discrepancies in the sample size, timing and amount of esketamine given, and the impact of influencing variables such as BMI, socioeconomic status, and the type of cesarean section procedure.

The aim of this study was to examine the preventative effects of a higher dose of esketamine infusion (1.5 mg/kg) as an adjunct therapy in PCIA on the incidence of PPD in Chinese women undergoing elective cesarean section. The risk of PPD was assessed by evaluating screening results, focusing specifically on individuals who scored 13 or higher on the Edinburgh Postnatal Depression Scale (EPDS). We also investigated the potential analgesic effects of esketamine on postoperative parturients in our study. Additionally, we assessed the levels of stress hormones, including free cortisol (FC), atrial natriuretic peptide (ANP), epinephrine (E), and norepinephrine (NE), as well as inflammation-inducing factors such as interleukin-6 (IL-6), C-reactive protein (CRP), and brain-derived neurotrophic factor (BDNF) in the blood. These markers have been shown to play a role in postpartum depression and the antidepressant effects of ketamine [20, 21].

## Methods

The current study was a prospective, double-blind, randomized controlled trial conducted at the First Hospital of Putian City in China from January 1, 2023, to September 31, 2023. The hospital sees approximately 2000 births annually, with 1000 of these being cesarean deliveries. The research obtained ethical approval (2022-026) from the Institutional Ethics Committee of the First Hospital of Putian City. Furthermore, it was registered with the Chinese Clinical Trial Registry (ID: ChiCTR2200067054) on December 26, 2022. Prior to the commencement of the trial, all participants provided informed consent.

A total of 246 pregnant women, classified as American Society of Anesthesiologists physical status II and aged between 19 and 44 years, underwent elective surgical procedures under spinal-epidural anesthesia as part of this study. The exclusion criteria included: (1) mental disorders; (2) serious obstetric complications or underlying medical conditions; (3) allergy to esketamine; and (4) ineligibility for intraspinal anesthesia.

**Fig. 1:**
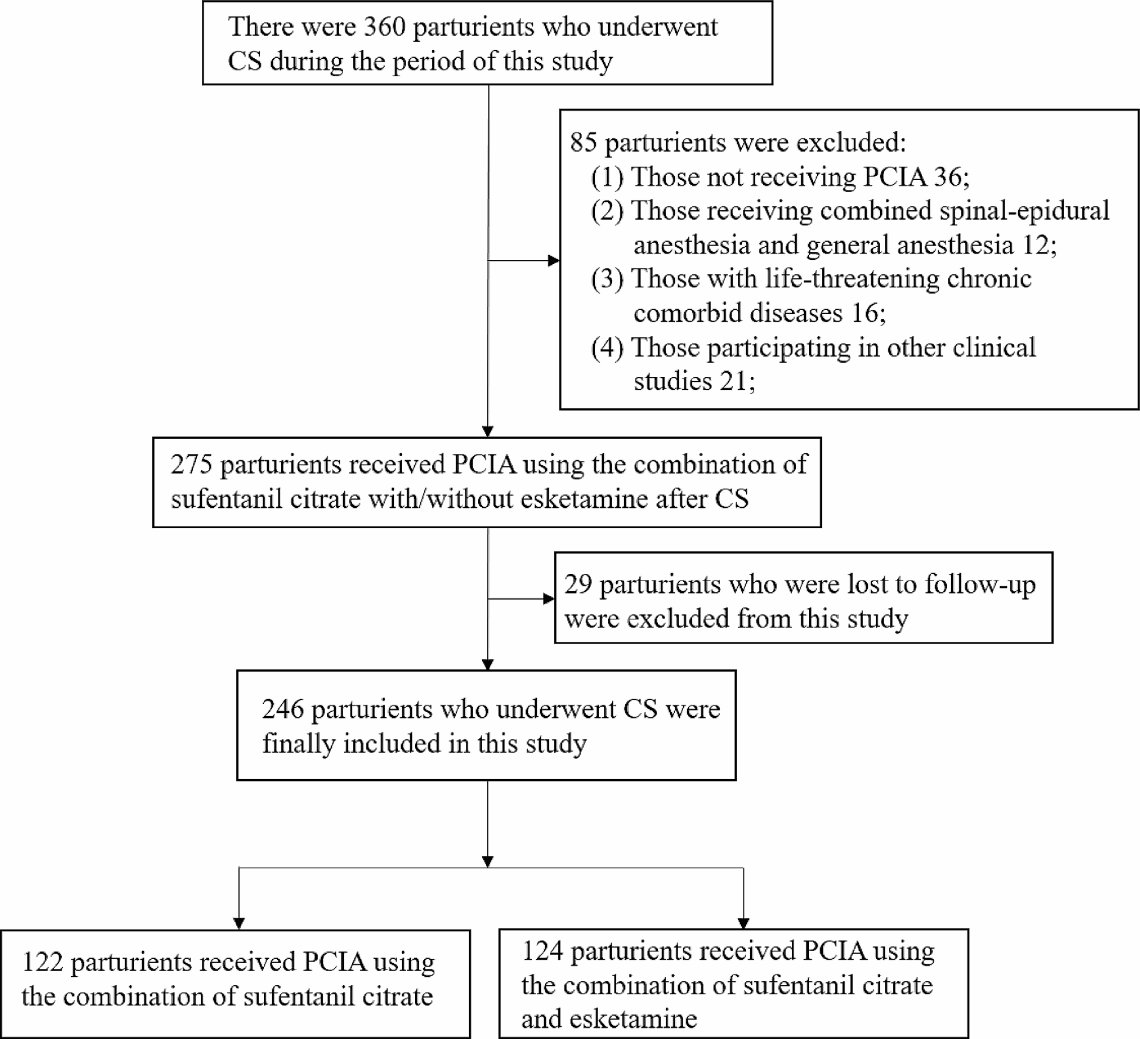
CONSORT flow diagram illustrating the study design and the inclusion/exclusion patient population flow. CONSORT Consolidated Standards of Reporting Trials. CS, cesarian section; PCIA, patient-controlled intravenous analgesia

Based on the initial trial findings, which included 20 participants in each group, the pre-test data indicated that the prevalence of PPD was 24% in the placebo group and 10% in the esketamine group. To achieve a statistical power of 80% and a significance level of 5%, the calculated sample size required for each group was 110 subjects. This calculation was conducted using the PASS 15.0 program from Stata Corp. LP, College Station, TX, USA. To accommodate a projected 10% participant dropout rate, it was determined that each arm of the study would need 123 participants. The recruitment flowchart, adhering to the Consolidated Standards of Reporting Trials, is provided in Fig. 1.

The subjects were randomized to two groups, namely the esketamine and the control groups, in a random manner, with an equal number of participants in each group. The esketamine group received esketamine, while the control group received normal saline. The process of simple randomization was performed utilizing IBM SPSS program version 26.0 (SPSS Inc., Chicago, IL, USA). This program produced random numbers to allocate participants to different groups. The pharmacist made syringes filled with esketamine or a solution of normal saline. The aforementioned items were enclosed into envelopes that were numbered in consecutive order and afterwards transported to the designated operation room. The nurse sequentially opened the envelopes in accordance with their numbered order. Every individual envelope was found to contain a syringe. The identity of the assigned treatment was kept undisclosed to patients, investigators, and study staff.

All females who experienced delivery had spinal anesthetic at the L3-L4 or L2-L3 and subjected to PCIA after operation. The administration of the anesthetic solution, consisting of 2 mL of 1% ropivacaine (Naropin, Astrazeneca AB. Sweden) and 1 mL of 10% glucose (Fresenius Kabi, Beijing, China), was performed by a qualified anesthesiologist. The dosage provided was 1 mL every 5 s, with the aim of attaining the maximum degree of sensory block at the T4-T6. Following the completion of the surgical procedure, all patients were given non-steroidal anti-inflammatory medicines in the form of a 40 mg IV dose of flurbiprofen (Beijing Taide Pharmaceutical Co., Ltd., Beijing, China). Additionally, preemptive analgesia was provided utilizing ultrasound-guided transversus abdominis plane block. The control group in the study included a combination of 2 µg/kg sufentanil (Yichang Humanwell Pharmaceutical Co., Ltd., Yichang China) and 10 mg tropisetron (Novartis Pharma, Beijing, China). On the other hand, the esketamine group received a combination of 2 µg/kg sufentanil, 10 mg tropisetron, and esketamine (No. 210425BL, Jiangsu Hengrui Pharmaceutical Co., Jiangsu, China) at a dosage of 1.5 mg/kg. These combinations were administered in a total volume of 100 mL. All participants were administered a continuous infusion at a baseline rate of 2 mL/h, as well as a 1 mL on-demand bolus with a 15 min lockout interval. The administration of the infusion initiated instantly after the closure of the skin incision and continued for two days. The EPDS questionnaire was administered to the subjects in a face-to-face interview conducted the day before to surgical preparation. The participants were contacted by telephone or WeChat in order to finish EPDS 42 days post-cesarean section, with the aim of diagnosing PPD. EPDS, a diagnostic instrument used for detecting PPD cases [22], has undergone translation into the Chinese language to facilitate the assessment of Chinese patients [23]. The EPDS comprises a set of 10 questions, with each item carrying a maximum score of three points. Consequently, the cumulative score attainable on the EPDS equals 30 points. In the present investigation, we classified EPDS scores equal to or more than 13 as indicative of postpartum depression [24].

The main objective of the research was to assess the occurrence of PPD risk 42 days after childbirth. This was accomplished by employing the EPDS administered by means of a web-based questionnaire. The secondary outcomes assessed in the study included cumulative sufentanil consumption, postoperative pain intensity, plasma levels of stress hormone levels and PPD inflammation-inducing factors and incidence of adverse events. The total amount of sufentanil consumed throughout certain time periods after the surgical procedure, including the intervals of 0–24 h, 24–48 h, and 0–48 h. Pain severity was evaluated utilizing the numeric rating scale (NRS) score, with pain scores at rest and throughout movements documented at 2, 4, 8, 12, 24, and 48 h after cesarean delivery. The enzyme-linked immunosorbent assay was employed to detect the plasma levels of FC, ANP, E, NE, CRP, IL-6, and BDNF on the day prior to preparation of surgery and on day 3 after surgery. The blood samples were obtained between the time frame of 6 to 7 a.m. Negative side effects throughout PCIA, like headache, nausea, vomiting, dizziness, drowsiness, and pruritus were reported. Adverse events were classified according to a recent definition (Common Terminology Criteria for adverse events-CTCAE, 2017). Demographic features, like weight, height, calculated BMI, age, gestational weeks, pregnancy complications, gravidity, parity, marital status, educational level, and EPDS scores, were obtained the day prior to the operation.

SPSS IBM statistical program package, version 26.0 (SPSS Inc., Chicago, IL), was employed to conduct Data analysis. Shapiro-Wilk test was utilized to detect the Normality assumptions for continuous variables. Statistical analysis was conducted for the measurement data, which included preoperative information such as age and BMI, EPDS scores, amount of sufentanil consumed, and plasma levels of FC, ANP, E, NE, CRP, IL-6, and BDNF. Normally distributed data were assessed for significance using independent sample t-tests, while non-normally distributed data were analyzed using the Kruskal-Wallis test. Unordered categorical data, such as gravidity, parity, gestational weeks, pregnancy complications, marital status, and educational level, as well as the incidence of PPD and adverse events, were evaluated using a chi-square test. The numerical rating scale (NRS) score was subjected to repeated measures analysis of variance (ANOVA), with multiple comparisons analyzed using Bonferroni adjustment. Results were considered statistically significant when the *p*-value was less than 0.05.

## Results

**Fig. 2:**
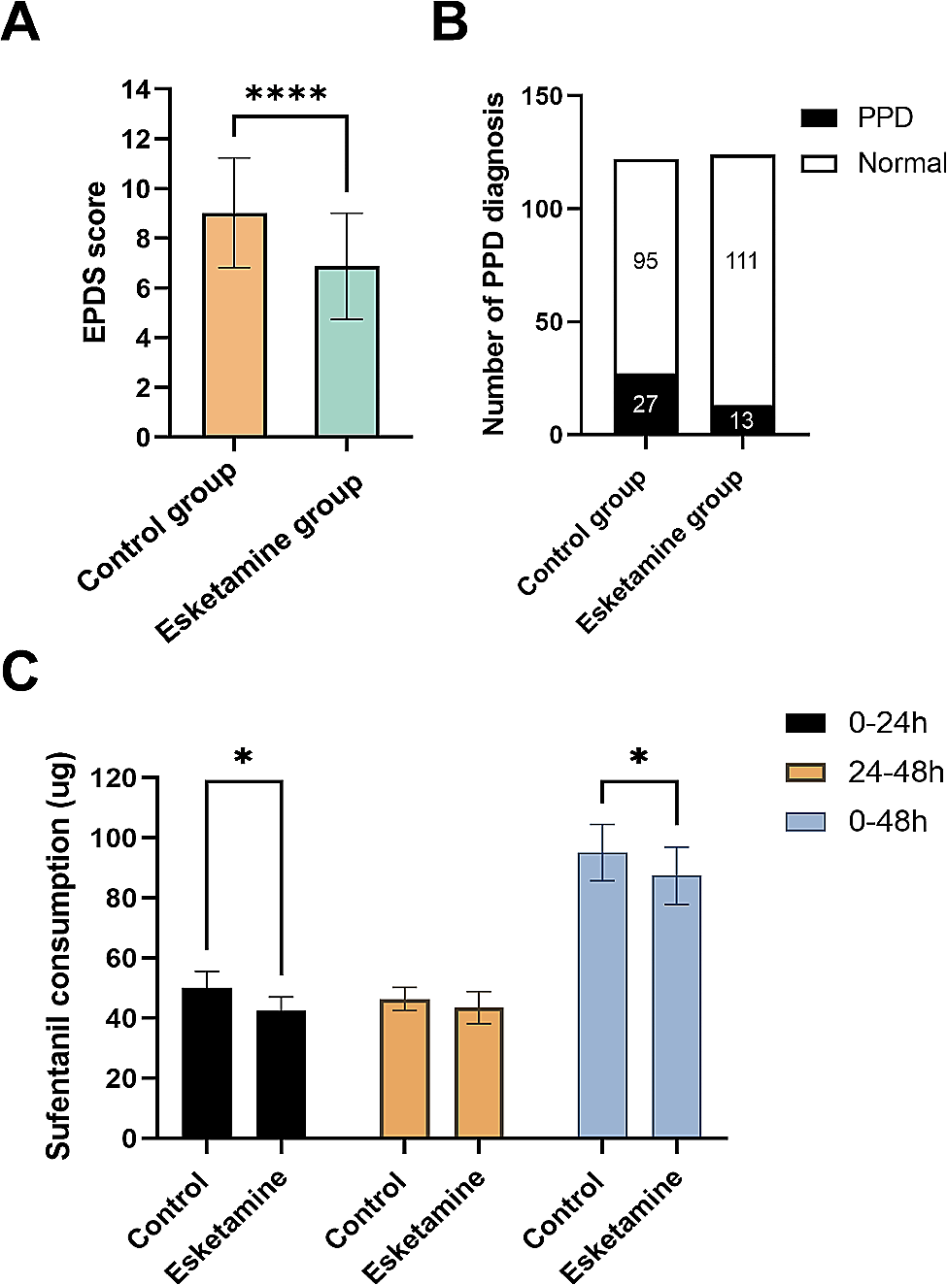
The risk of PPD and cumulative sufentanil consumption up to 48 h postoperatively. **A** The EPDS scores of patients between the control and esketamine groups. **B** the incidence of PPD at 42 days after surgery. **C** cumulative sufentanil consumption up to 48 h postoperatively. Data were presented as mean ± SD. ****Indicates that the scores on the EPDS were significantly lower in the esketamine group than in the control group (*P* < 0.0001). *Indicates a significant decrease in sufentanil consumption in the esketamine group compared to the control group during 0–24 h, and 0–48 h postoperatively (*P* < 0.05). PPD, postpartum depression; EPDS, Edinburgh Postnatal Depression Scale

A total of 360 parturients were initially enrolled in the study between January 1, 2023, to September 31, 2023. However, 36 of them declined to receive PCIA after cesarean delivery, 12 underwent combined spinal-epidural anesthesia and general anesthesia, 16 had life-threatening chronic comorbid diseases, 21 were already participating in other clinical studies, and 29 were lost to follow-up. As a result, the final study included a total of 246 cases (Fig. 1). Table 1 presents a comprehensive overview of the patients’ demographic and clinical characteristics. No statistically significant differences were observed between the two groups (Table 1).The incidence of depression was found to be significantly lower in the group that received esketamine compared to the control group 42 days after cesarean Sect. (22.2% and 10.7%, respectively, *P* = 0.015) (Fig. 2). The levels of EPDS scores were similar between the esketamine group and the control group before the operation (3.6 ± 3.0 vs. 4.0 ± 2.1, *P* = 0.23). However, there were significant differences in EPDS scores between the two groups at 42 days postpartum (6.87 ± 2.14 vs. 9.02 ± 2.21, *P* < 0.0001) (Fig. 2).

**Table 1:** Preoperative data of the included pregnant women

Variables	Control group	Esketamine group	*P* value
Number	122	124	
Age (year), mean ± SD	27.7 ± 4.8	28 ± 3.1	0.56
Pre-pregnancy BMI (kg/m2), mean ± SD	24.1 ± 3.1	24.5 ± 4.7	0.43
Gestational weeks, n (%)			0.85
Full term Preterm Post term	101 (82.77) 14 (11.48) 7 (5.74)	106 (85.48) 12 (9.67) 6 (4.84)	
Pregnancy complications, n (%)			
Gestational diabetes Gestational hypertension Gestational Low thyroid	13 (10.66) 21 (17.21) 32 (26.23)	12 (9.68) 24 (19.35) 35 (28.23)	0.84 0.74 0.78
Gravidity, n (%)			0.80
0 ≥1	47 (38.52) 75 (61.48)	50 (40.32) 74 (59.68)	
Parity, n (%)			0.80
0 ≥1	61 (50) 61 (50)	65 (52.42) 59 (47.58)	0.80
Marital status, n (%)			0.61
Married Divorced Unmarried	121 (99.18) 0 (0) 1 (0.82)	122 (98.39) 1 (0.81) 1 (0.81)	
Educational level, n (%)			0.43
Master or above Bachelor or below	43 (35.25) 79 (64.75)	50 (40.32) 74 (59.68)	
Stress hormone levels, mean ± SD			
Free cortisol (FC) (nmol/L)	261.51 ± 55.32	251.98 ± 62.23	0.21
Atrial natriuretic peptide (ANP) (ng/mL)	0.54 ± 0.25	0.59 ± 0.19	0.08
Epinephrine (E) (ng/L)	37.09 ± 4.32	35.92 ± 5.39	0.06
Norepinephrine (NE) (ng/L)	159.82 ± 32.21	155.16 ± 43.13	0.34
Inflammation-inducing factors, mean ± SD			
C-reactive protein (CRP) (mg/dl)	0.32 ± 0.11	0.34 ± 0.09	0.12
Interleukin-6 (IL-6) (pg/mL)	2.30 ± 0.7	2.11 ± 1.01	0.11
Brain-derived neurotrophic factor (BDNF) (pg/mL)	1.31 ± 0.09	1.29 ± 0.12	0.14
Edinburgh Postpartum Depression Scale, mean ± SD	4.0 ± 2.1	3.6 ± 3.0	0.23

SD, Standard Deviation

**Fig. 3:**
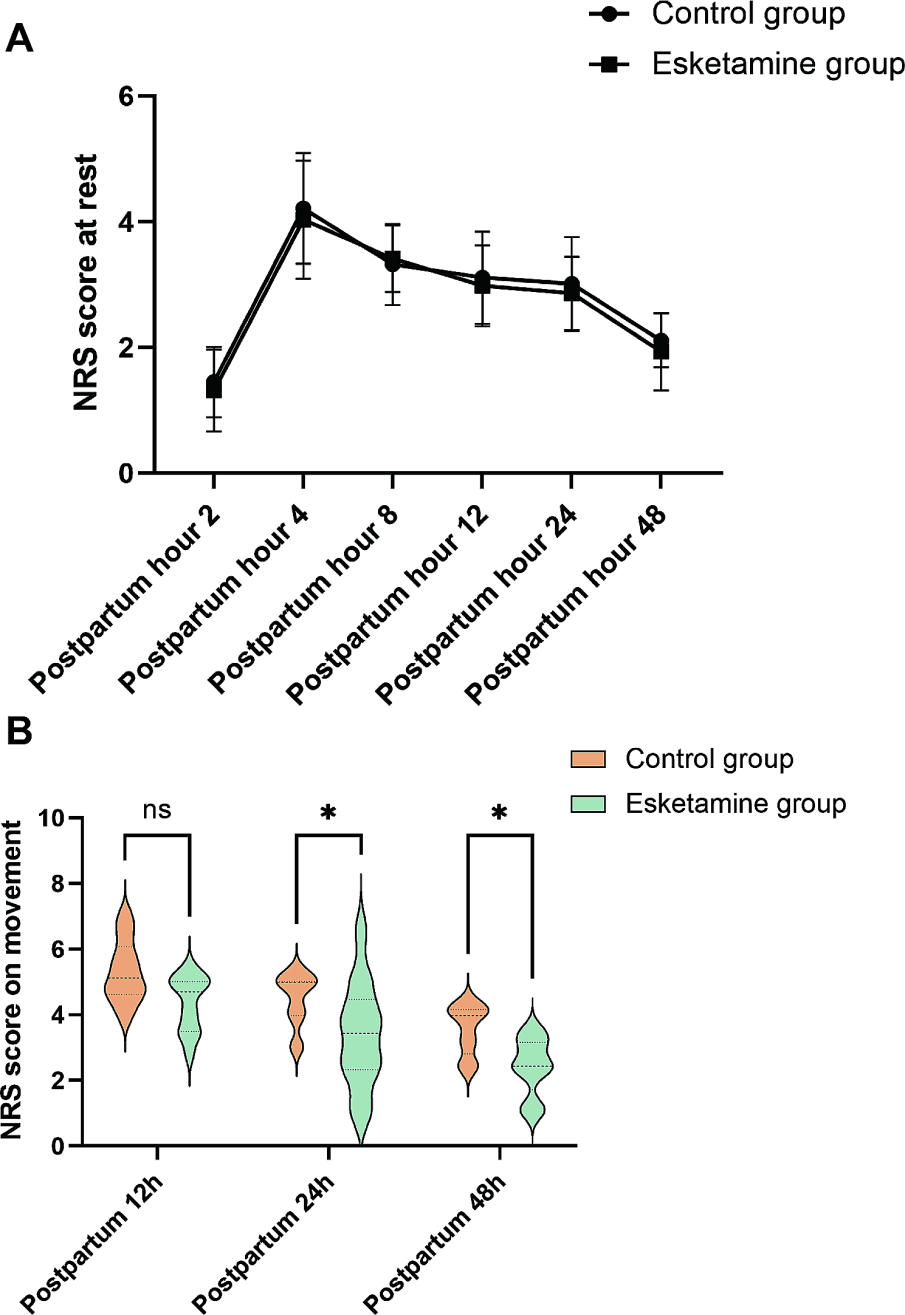
Comparison of NRS scores between the control and esketamine groups at rest and during exercise at the same time point. **A** Resting NRS scores between the two groups at 2, 4, 8, 12, 24 and 48 h postpartum. **B** NRS scores during exercise between the two groups at 12, 24 and 48 h postpartum. *Indicates a significant decrease in NRS scores at rest in the esketamine group compared to the control group (*P* < 0.05). **Indicates *P* < 0.01, ***P < 0.001, and ****P < 0.0001. NRS, numerical rating scale

The consumption of sufentanil in the esketamine group was significantly lower than that in the control group on the first day (42.5 ± 4.58 µg vs. 50.15 ± 5.47 µg, *P* = 0.04) and the second day (87.40 ± 9.51 µg vs. 95.10 ± 9.36 µg, *P* = 0.04) after surgery. However, there was no significant difference observed during the 24–48 h postpartum period (43.50 ± 5.34 µg vs. 46.40 ± 3.86 µg, *P* = 0.71) (Fig. 2). NRS scores at rest remained unchanged in the esketamine group at 2, 4, 8, 12, 24, and 48 h after cesarean Sect. (1.11 ± 0.65 vs. 1.31 ± 0.56, *P* = 0.49; 4.03 ± 0.94 vs. 4.21 ± 0.88, *P* = 0.20; 3.41 ± 0.53 vs. 3.32 ± 0.64, *P* = 0.88; 2.98 ± 0.64 vs. 3.11 ± 0.73, *P* = 0.57; 2.86 ± 0.58 vs. 3.01 ± 0.75, *P* = 0.40; 1.91 ± 0.62 vs. 2.11 ± 0.43, *P* = 0.20, respectively). However, significant differences in movement were observed between the two groups at 24 and 48 h after cesarean Sect. (3.39 ± 1.57 vs. 4.50 ± 0.80, *P* = 0.02; 2.43 ± 0.87 vs. 3.56 ± 0.76, *P* = 0.02), but not at 12 h (4.35 ± 0.86 vs. 5.32 ± 0.95, *P* = 0.06) (Fig. 3).

**Fig. 4:**
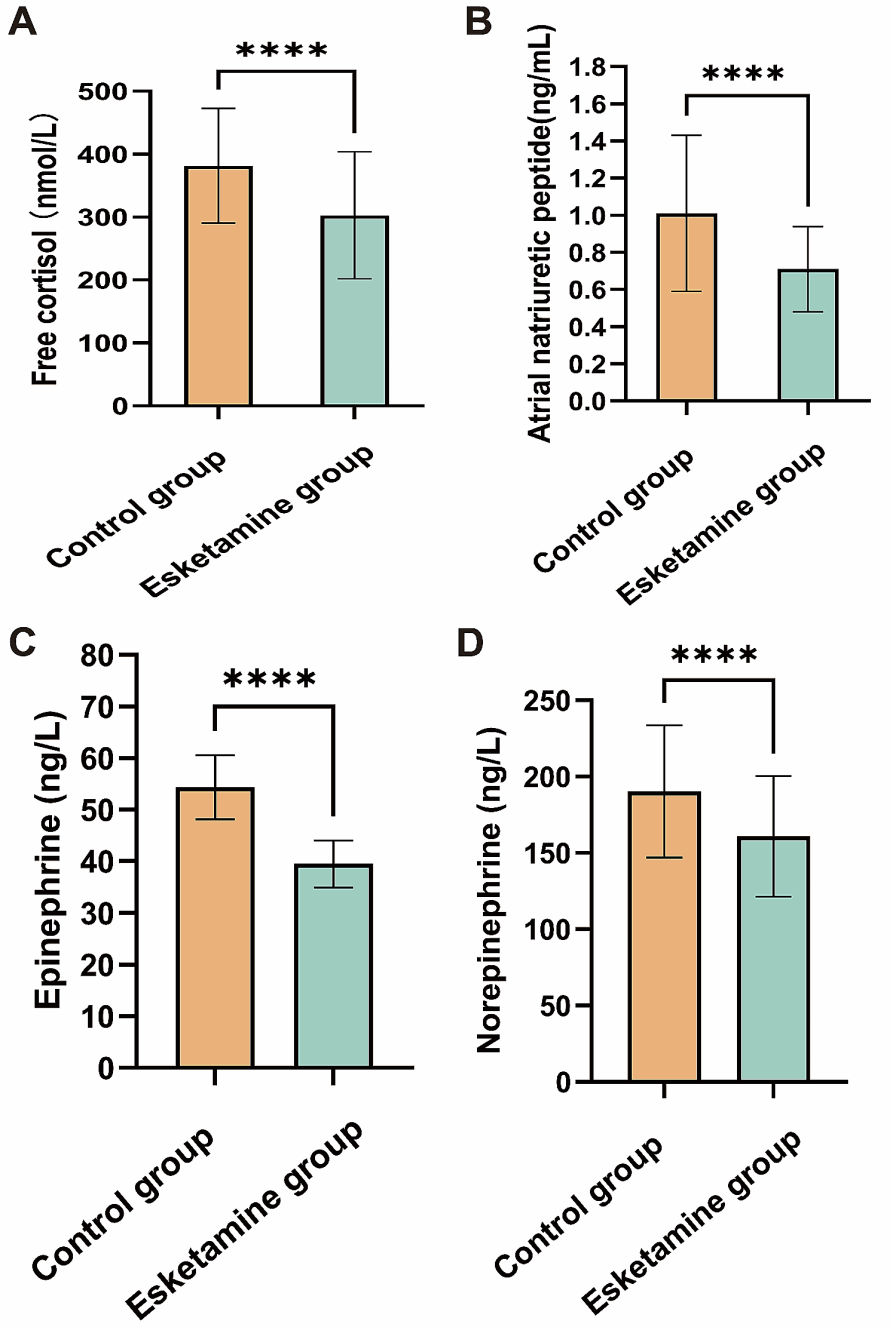
Stress hormone expression levels between control and esketamine groups on postoperative day 3. **A** FC expression levels between the two groups on postoperative day 3. **B** comparison of the expression levels of ANP between the two groups on the 3rd post-operative day. **C** expression levels of Ebetween the two groups on postoperative day 3. **D** NE expression levels between the two groups on postoperative day 3. ****Indicates a significant decrease in FC, ANP, E and NE in the esketamine group compared to the control group (*P* < 0.0001). FC, free cortisol; ANP, atrial natriuretic peptide; E, epinephrine; NE, norepinephrine

**Fig. 5:**
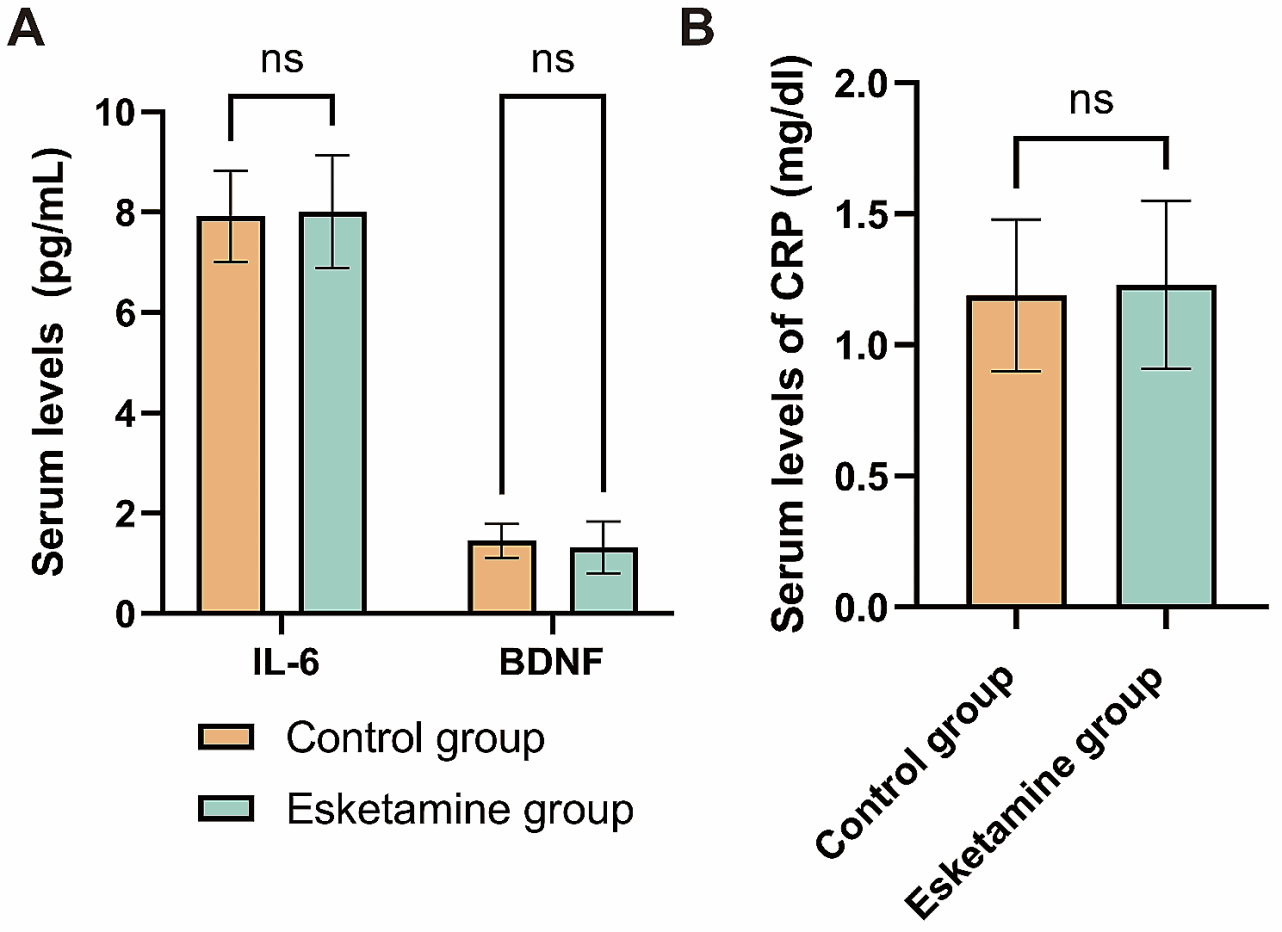
Expression levels of the inflammation inducing factors of PPD were compared between the control group and the esketamine group on the 3rd post-operative day. **A** expression levels of IL-6 and BDNF between the two groups on postoperative day 3. **B** CRP expression levels between the two groups on day 3 after surgery. PPD, postpartum depression; IL-6, interleukin-6; BDNF, brain-derived neurotrophic factor; CRP, C-reactive protein

The levels of Serum FC and ANP were significantly lower in the esketamine group compared to the control group (FC: 302 ± 101 vs. 382 ± 91 nmol/L, *P*<0.0001; ANP: 0.71 ± 0.23 vs. 1.01 ± 0.42 ng/mL, *P*<0.0001). Additionally, the E and NE levels were also significantly decreased in the esketamine group compared to the control group (E: 39.47 ± 4.58 vs. 54.32 ± 6.19 ng/L, *P*<0.0001; NE: 160.84 ± 39.45 vs. 190.31 ± 43.29 ng/L, *P*<0.0001) (Fig. 4). There were no statistically significant differences in the plasma concentrations of CRP and IL-6 between the two groups on the third day following the surgical procedure (CRP: 1.23 ± 0.32 vs. 1.19 ± 0.29 mg/ml, *P* = 0.31; IL-6: 8.01 ± 1.12 vs. 7.92 ± 0.91 pg/mL, *P* = 0.49, respectively). Similarly, there were no statistically significant variations observed in the plasma concentrations of BDNF between the two groups (1.32 ± 0.62 vs. 1.45 ± 0.44 pg/mL, *P* = 0.06) (Fig. 5).

The most frequent side effects observed in the placebo group during the surgical procedure were nausea and vomiting (27.42%), dizziness (16.94%), pruritus (8.84%), and drowsiness (2.41%). In the Esketamine group, the predominant side effects were nausea and vomiting (25.41%), dizziness (22.95%), pruritus (6.56%), and drowsiness (0.82%). However, there were no statistically significant differences in the occurrence of adverse events between the two groups (*P* > 0.05, Table 2).

**Table 2:** Analgesia-related adverse events, n (%)

Variables	Control group	Esketamine group	*P* value
Nausea and vomiting	31(25.41)	34(27.42)	0.77
Dizziness	28(22.95)	21(16.94)	0.27
Pruritus	8(6.56)	6(8.84)	0.59
Drowsiness	1(0.82)	3(2.41)	0.62

## Discussion

The findings of this randomized controlled trial demonstrated that postoperative esketamine treatment was effective in reducing the risk of PPD in women undergoing elective caesarean section. Additionally, esketamine was found to enhance postoperative pain management and decrease sufentanil usage following elective caesarean section, without a significant increase in psychological adverse events. Furthermore, esketamine was shown to significantly decrease the release of stress-related hormones, though no significant changes were observed in inflammation-inducing factors of PPD.

A recent randomized clinical trial suggested that intravenous infusion of esketamine may reduce the incidence of PPD in women after caesarean Sect. [25]. However, a study conducted by Liu et al. aimed to investigate the effects of postoperative administration of esketamine on the incidence of PPD in women undergoing elective caesarean section. The researchers administered 0.25 mg/kg of esketamine intraoperatively and 1.25 mg/kg via patient-controlled intravenous analgesia (PCIA). Their results showed that this intervention did not reduce the risk of PPD in this particular population [16]. Based on the available data, it appears that the observed difference between our study and the previous study may be attributed to the difference in dose (1.5 mg/kg in our study) and timing (as an adjunct to PCIA in our study) of esketamine.

The findings of the investigation suggest that postoperative analgesia with esketamine may have a more noticeable impact on pain relief during movement for up to 48 h after surgery. However, there was no significant difference in pain relief at rest. These results are consistent with earlier studies [16, 18, 25], which have shown that the use of esketamine through intravenous infusion as an adjunct to PCIA led to a decrease in the total amount of pain medication consumed during the first 24 and 48 h after surgery. Studies have also shown that esketamine can reduce opioid consumption after a caesarean Sects. [26, 27]. Recent preclinical studies have provided evidence that the mu-opioid receptor contributes to the pharmacological effects of esketamine [28]. Given its interaction with the opioid system, it is suggested that the administration of esketamine may hold potential for reducing opioid use in women following elective caesarean section.

The stress response is a common complication of surgery and anesthesia, triggering the hypothalamic-pituitary-adrenal cortex system and leading to increased levels of stress hormones [29]. In our study, we found that the levels of certain stress-related factors were lower in the esketamine group compared to the control group, indicating that esketamine could effectively reduce postoperative stress and trauma. Furthermore, a systematic review demonstrated that markers of inflammation such as CRP and IL-6 are indicative of PPD [30]. Elevated levels of these markers upon admission were shown to be predictive of PPD within a six-month period. Additionally, a deficiency in BDNF has been identified as a significant factor in perinatal depression [31, 32]. However, our study did not find significant differences in the plasma concentrations of these markers between the groups, despite the reduction in PPD incidence in the esketamine group at 42 days postoperatively. This suggests that the levels of markers associated with PPD at 3 days postoperatively may not accurately reflect the risk of medium and long-term PPD.

This investigation has restrictions. Firstly, the study was conducted on Han Chinese women undergoing caesarean section. Therefore, more multicenter studies with larger sample sizes are needed to investigate the effects of esketamine on postpartum depression, including diverse ethnic populations and different modes of delivery. Secondly, we did not use the American Psychiatric Association’s DSM-5 to diagnose PPD, although a threshold EPDS score of 12/13 has been validated [22]. In addition, we did not separately consider EPDS item 10 to identify individuals at risk. Thirdly, our study used an esketamine dose of 1.5 mg/kg in PCIA, which differs from the dosage used in the treatment of depression [17, 33]. Therefore, further investigation is needed to determine whether higher or lower doses of esketamine could be more effective and safer in the prevention and treatment of PPD. Fourthly, PPD can manifest within a range of timeframes after childbirth, including as early as 6 weeks postpartum or as late as 36 months after delivery [2, 3]. Our patients were followed for 6 weeks to assess the impact of esketamine on PPD. The long-term effects of the current intervention remain to be determined.

In conclusion, our investigation has shown that postoperative injection of esketamine can effectively reduce the risk of PPD in females undergoing elective cesarean section. This was accompanied by a significant decrease in the concentration of stress-associated hormones in the esketamine groups. Additionally, we observed that esketamine led to lower pain scores during movement and reduced the need for opioid consumption following cesarean section, without causing a significant increase in psychological adverse events. However, further large-scale randomized, double-blind clinical studies are needed to determine the optimal timing, route, and dosage of esketamine administration for the prevention of PPD.

## Data Availability

The datasets used and/or analysed during the current study are available from the corresponding author on reasonable request.

## Abbreviations

PPD: Postpartum depression
PCIA: Patient-controlled intravenous analgesia
EPDS: Edinburgh postnatal depression scale
NRS: Numerical rating scale
NMDAR: N-methyl-D-aspartate receptor
IV: Intravenous
BMI: Body mass index
FC: Free cortisol
ANP: Atrial natriuretic peptide
E: Epinephrine
NE: Norepinephrine
IL-6: Interleukin-6
CRP: C-reactive protein
BDNF: Brain-derived neurotrophic factor
ANOVA: Repeated measures analysis of variance
